# Functional analysis of BPSS2242 reveals its detoxification role in *Burkholderia pseudomallei* under salt stress

**DOI:** 10.1038/s41598-020-67382-y

**Published:** 2020-06-26

**Authors:** Kamonwan Chamchoy, Pornpan Pumirat, Onrapak Reamtong, Danaya Pakotiprapha, Ubolsree Leartsakulpanich, Usa Boonyuen

**Affiliations:** 1 Department of Molecular Tropical Medicine and Genetics, Faculty of Tropical Medicine, Mahidol University, Bangkok, 10400 Thailand; 2 Department of Microbiology and Immunology, Faculty of Tropical Medicine, Mahidol University, Bangkok, 10400 Thailand; 3 Department of Biochemistry, Faculty of Science, Mahidol University, Bangkok, 10400 Thailand; 4 Center for Excellence in Protein and Enzyme Technology, Faculty of Science, Mahidol University, Bangkok, 10400 Thailand; 5 National Center for Genetic Engineering and Biotechnology, National Science and Technology Development Agency, Pathumthani, 12120 Thailand

**Keywords:** Biochemistry, Biophysics, Genetics, Microbiology, Molecular biology

## Abstract

A *bpss2242* gene, encoding a putative short-chain dehydrogenase/oxidoreductase (SDR) in *Burkholderia pseudomallei*, was identified and its expression was up-regulated by ten-fold when *B. pseudomallei* was cultured under high salt concentration. Previous study suggested that BPSS2242 plays important roles in adaptation to salt stress and pathogenesis; however, its biological functions are still unknown. Herein, we report the biochemical properties and functional characterization of BPSS2242 from *B. pseudomallei*. BPSS2242 exhibited NADPH-dependent reductase activity toward diacetyl and methylglyoxal, toxic electrophilic dicarbonyls. The conserved catalytic triad was identified and found to play critical roles in catalysis and cofactor binding. Tyr162 and Lys166 are involved in NADPH binding and mutation of Lys166 causes a conformational change, altering protein structure. Overexpression of BPSS2242 in *Escherichia coli* increased bacterial survival upon exposure to diacetyl and methylglyoxal. Importantly, the viability of *B. pseudomallei* encountered dicarbonyl toxicity was enhanced when cultured under high salt concentration as a result of BPSS2242 overexpression. This is the first study demonstrating that BPSS2242 is responsible for detoxification of toxic metabolites, constituting a protective system against reactive carbonyl compounds in *B. pseudomallei.*.

## Introduction

*Burkholderia pseudomallei* is the causative agent of melioidosis, a severe infectious disease endemic in Southeast Asia and northern Australia and is increasingly recognized in non-endemic areas, including USA, India, Southern China, Brazil and Malawi^1,2^. *B. pseudomallei* is an environmental saprophytic bacterium that inhabits soil and water and is constantly exposed to diverse environments. The pathogen was reported to possess the ability to survive in hostile environments, including lack of nutrients, a wide range of temperatures, and exposure to acidic, dry, and oxidative environments^3,4^.

In Thailand, melioidosis is common in the northeastern region, with a reported fatality rate as high as 40%^5^. A high incidence of the disease is associated with the presence of *B. pseudomallei* in the soil where the electrical conductivity is in the range of 4–100 dS/m, which is significantly higher than soil in other regions (2 dS/m)^6,7^. The viability and culturability of the pathogen were reported for up to 90 days after exposure to saline soil^8,9^. *B. pseudomallei* could survive when cultured overnight in medium supplemented with up to 2.5% *(w/v)* NaCl, indicating its persistence to salt stress^4^. Alteration of several metabolic enzymes, transcription/translation regulators, chaperones, drug-resistant proteins, and potential virulence factors was detected in *B. pseudomallei* grown in salt-rich medium^10^. Moreover, it was shown that the salt-exposed pathogen invaded a lung epithelial cell line A549 more efficiently and exhibited significantly greater resistance to ceftazidime, an effective antibiotic used to treat melioidosis^10,11^. This suggests the ability of *B. pseudomallei* to survive and adapt under salt stress.

Global transcriptional analysis demonstrated that *B. pseudomallei* responded to salt stress by modulating the transcription of several genes^11^. Interestingly, the *bpss2242* gene, encoding a putative short chain dehydrogenase/oxidoreductase (SDR), was up-regulated ten-fold when *B. pseudomallei* K96243 was grown in NaCl-supplemented medium^11^. SDR is a large protein superfamily, in which NAD(P)(H)-dependent oxidoreductases are the majority of enzymes^12^. SDRs play important biological roles in many organisms, because the substrates of several SDRs are known to serve as crucial biological molecules in cells^12,13^. In *B. pseudomallei,* BPSS2242 was demonstrated to play important roles in bacterial invasion and intracellular survival. The ability to invade the lung epithelial cell line A549 and to survive in the macrophage in the initial stage of infection was impaired in the BPSS2242 deleted mutant (△BPSS2242). Additionally, the glucose dehydrogenase (GDH) activity in the bacterial crude extract of the △BPSS2242 mutant was 15-fold lower than the wild type (WT)^14^. These suggested that BPSS2242 is involved in the survival, adaptation and pathogenesis of *B. pseudomallei*. However, the biological function of BPSS2242 in *B. pseudomallei* has not been reported before.

To investigate the function and biological role of BPSS2242 from *B. pseudomallei*, we thoroughly characterized this protein. BPSS2242 was cloned, expressed, purified to homogeneity and its biophysical and biochemical properties, including oligomeric state, cofactor and substrate specificity, thermal stability, and kinetic parameters were determined. In order to obtain more details of enzyme catalysis, site-directed mutagenesis of catalytic residues was performed and the roles of each residue on catalysis were demonstrated. Additionally, the biological role of BPSS2242 upon exposure to toxic dicarbonyl compounds was assessed in *E. coli* expressing BPSS2242 and in *B. pseudomallei*.

## Results

### Sequence analysis reveals a unique character of BPSS2242

**Figure 1:**
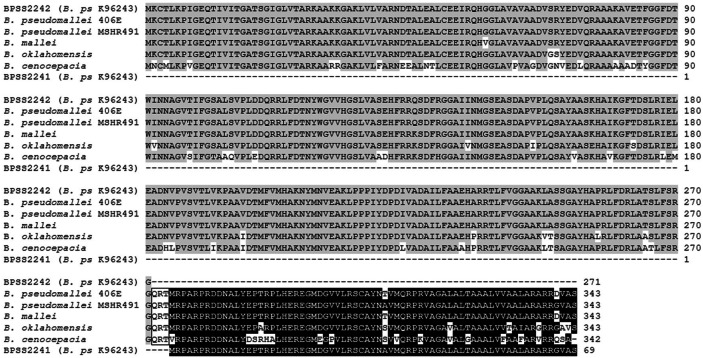
Multiple sequence alignments of BPSS2242 and BPSS2241 from *B. pseudomallei* K96243 and SDRs from other pathogenic *Burkholderia*. Homologs of BPSS2242 and BPSS2241 are shaded in gray and black, respectively. Box indicates additional amino acids found in other isolates.

The *bpss2242* gene (NCBI Reference Sequence: YP_112245.1) locates on chromosome 2 of *B. pseudomallei* isolate K96243 and encodes a putative 271-amino acid SDR with a predicted molecular weight (MW) of 28.9 kDa. BLAST analysis of *bpss2242* against the database of *Burkholderia* (https://www.burkholderia.com) revealed high sequence identity among the pathogenic strains (Supplementary Fig. S1). Interestingly, homologs of *bpss2242* contain additional sequence at the 3′-end, which is different from *B. pseudomallei* isolate K96243 used in this study. This additional sequence was identified as *bpss2241* (NCBI Reference Sequence: YP_112244.1), a locus downstream of *bpss2242* in *B. pseudomallei* isolate K96243. At amino acid level, BPSS2242 and BPSS2241 are linked by three amino acids (Glu, Arg and Thr) into a single protein in other *B. pseudomallei* isolates (Fig. 1), raising the possibility that BPSS2242 and BPSS2241 may function together. If they are separated into two proteins, cellular function of BPSS2242 may require BPSS2241 counterpart. To elucidate whether BPSS2241 is involved in catalytic activity of BPSS2242, the recombinant BPSS2241 and a single protein containing both BPSS2242 and BPSS2241 (BPSS2242 + 41) were also constructed and investigated.

**Figure 2:**
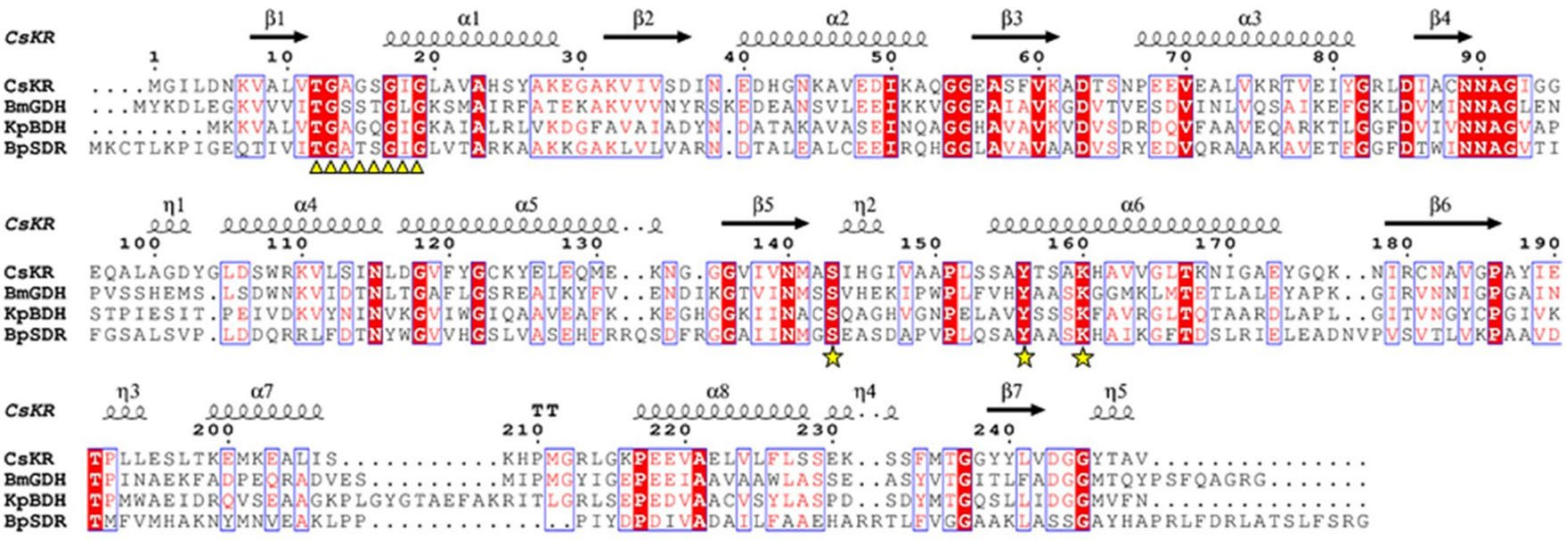
Multiple sequence alignments of BPSS2242 and other members of SDR superfamily. The alignment was performed using BioEdit program and rendered by ESPript. The secondary structure of CsKR was shown above the alignment. The consensus sequence of NAD(P)(H)-binding motif and catalytic triad are highlighted by triangles and stars, respectively.

Furthermore, sequence analysis reveals that BPSS2242 shares approximately 30–35% sequence identity with other members in SDR superfamily, including ketone reductase from *Chryseobacterium sp.* CA49 (CsKR; PDB ID: 5X8H), GDH from *Bacillus megaterium* (BmGDH; PDB ID: 1GCO), and butanediol dehydrogenase from *Klebsiella pneumoniae* (KpBDH; PDB ID: 1GEG). BPSS2242 also possesses the common features of SDR superfamily, characterized by the Gly rich coenzyme binding motif (TGxxxGxG), the active site motif (YxxxK), and the conserved catalytic triad (Ser149, Tyr162, and Lys166) (Fig. 2).

### Characterization of the recombinant *B. pseudomallei* SDRs (BpSDRs)

**Figure 3:**
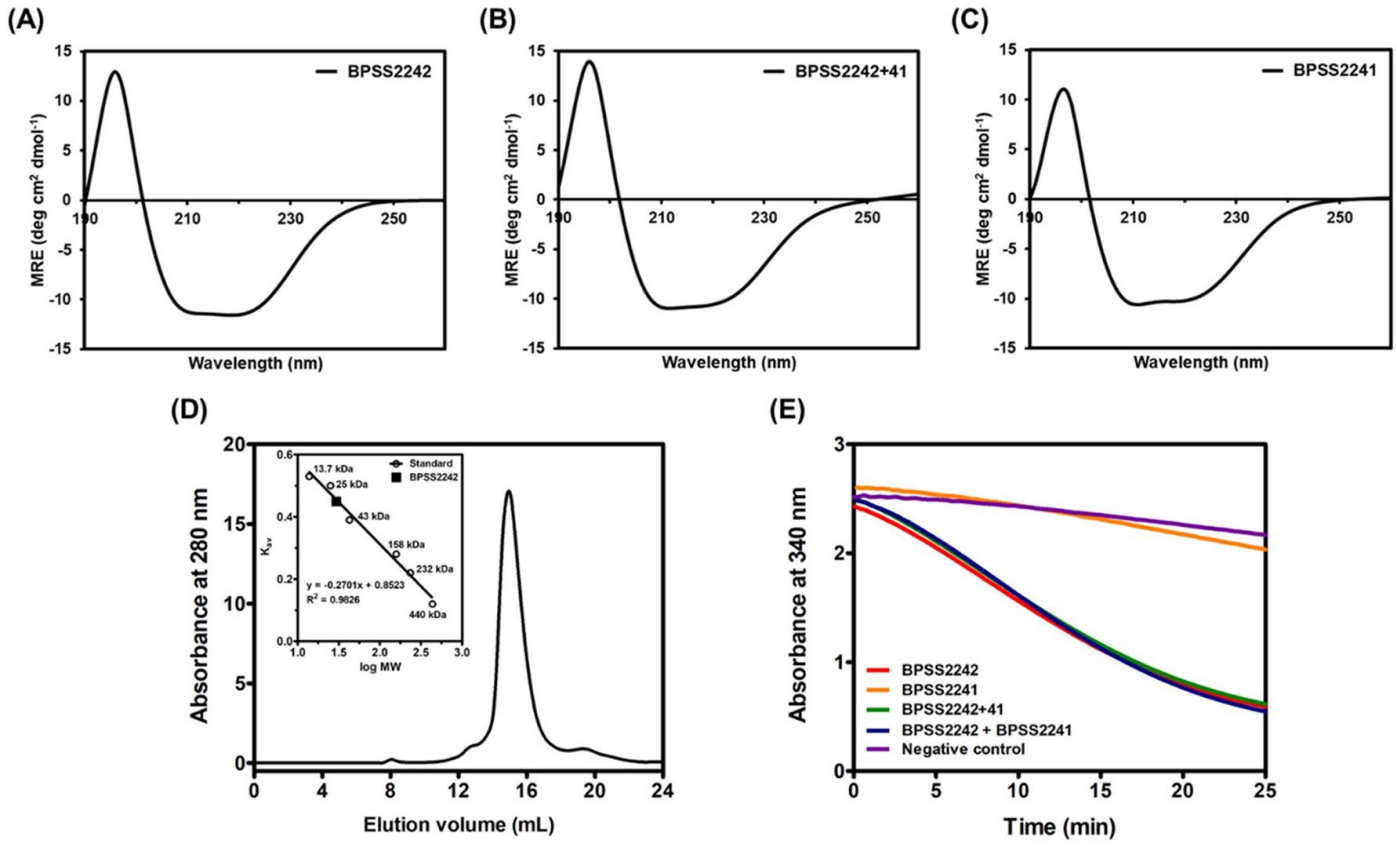
Analyses of purified recombinant proteins. CD spectra of (**A**) BPSS2242, (**B**) BPSS2242 + 41, and (**C**) BPSS2241. (**D**) The standard curve and gel filtration chromatogram for determining native state of BPSS2242. (**E**) Diacetyl reductase assay of BpSDRs. Negative control is the reaction without enzyme.

Gene cloning, protein expression and purification of BPSS2242, BPSS2241, and BPSS2242 + 41 were described in supplementary information. Agarose gel electrophoresis of *bpss2242*, *bpss2241* and *bpss2242* + *41* is shown in Supplementary Fig. S2. BPSS2242 was expressed from pET23a-*bpss2242* with a C-terminal His-tag while BPSS2241 and BPSS2242 + 41 were expressed with an N-terminal His-tag from pET28a*-bpss2241* and pET28a-*bpss2242* + *41*, respectively. SDS-PAGE analysis of purified recombinant proteins is shown in Supplementary Fig. S3. To ensure that the folding of purified recombinant proteins is correct, secondary structures of these recombinant proteins were determined by circular dichroism (CD). CD spectra of all purified proteins showed negative peaks at 208 and 222 nm and a positive peak at 193 nm, which are a characteristic of α-helical protein (Fig. 3A–C). The CD data were further analyzed using the CDSSTR program with the reference data set SMP56, providing the relative contents of α-helix, β-sheet, turn and coil regions^15^. The secondary structure contents of each purified protein are shown in Table 1. It is worth mentioning that BPSS2241 contains greater α-helix content than BPSS2242 and BPSS2242 + 41. To determine the oligomeric state of BPSS2242, size exclusion chromatography was carried out. A single elution peak corresponding to a molecular mass of 29.6 kDa was observed (Fig. 3D), indicating that native BPSS2242 exists primarily as a monomer in 20 mM Tris–HCl pH 8.0 containing 500 mM NaCl.

**Table 1:** Secondary structure contents of BpSDRs.

Construct	Composition of secondary structure (%)			
	α-Helix	β-Sheet	Turn	Coil
BPSS2242	60	15	13	12
BPSS2242 + 41	60	15	13	12
BPSS2241	72	8	9	11

### Enzyme activity of the recombinant BpSDRs

To determine the enzyme activity of BpSDRs, various substrates, including sugars, alcohols, steroid, aldehydes, ketones and fatty acid, were screened (Tables 2, 3) based on previous reports^16–18^. BPSS2242 exhibited reductase activity toward diacetyl and methylglyoxal when NADPH was used as a cofactor. Compared with diacetyl, 20% activity was detected for methylglyoxal. Considering oxidation reactions, BPSS2242 showed marginal activities with glucose, galactose, fructose, and xylose when NAD^+^ was present. Purified BPSS2242 + 41 was also subjected to substrate screening in which it showed similar activities to those observed for BPSS2242. As shown in Fig. 3E, the activity of BPSS2242 + 41 for the reduction of diacetyl was comparable to that of purified BPSS2242 alone and BPSS2242 mixed with BPSS2241 counterpart. It is noted that the activity of purified BPSS2241 alone is comparable to that of the negative control, indicating that BPSS2241 is not involved in catalysis. However, the biological role of BPSS2241 is still unknown. Since BPSS2241 is not important for enzyme catalysis, further enzymatic characterizations were carried out focusing on BPSS2242.

**Table 2:** Substrates used in screening for reduction reaction of BPSS2242.

Type of substrate	Substrate	Enzyme activity (µM min^−1^ mg^−1^)
Monoaldehydes/ketones	Acetone	na
	Butanone	na
	Formaldehyde	na
	Acetaldehyde	na
	Butyraldehyde	na
	Propionaldehyde	na
	Acetoin	na
Uncharged dicarbonyl	Glyoxal	na
	Methylglyoxal	18.61^a^
	Diacetyl	92.21^a^
Charged dicarbonyl	Pyruvic acid	na
	Oxaloacetic acid	na
Non-vicinal dicarbonyls	Acetylacetone	na
	3, 5-Heptanedione	na
Fatty acid	Crotonyl-CoA	na

The reaction mixture contained 20 mM sodium phosphate (pH 6.5), 200 µM NAD(P)H and various concentrations of substrate (0.1–20 mM).

*na* no activity.

^a^NADPH was used as a cofactor.

**Table 3:** Substrates used in screening for oxidation reaction of BPSS2242.

Type of substrate	Substrate	Enzyme activity (µM min^−1^ mg^−1^)
Monosaccharides	α-Glucose	1.14^a^
	Galactose	0.34^a^
	Fructose	0.82^a^
	Xylose	3.33^a^
	Mannose	na
Disaccharides	Maltose	na
	Sucrose	na
	Lactose	na
	Trehalose	na
	Maltotriose	na
Aliphatic alcohols	Methanol	na
	Ethanol	na
	1-Propanol	na
	1-Butanol	na
	1-Hexanol	na
	1-Octanol	na
Aromatic alcohol	Benzyl alcohol	na
Polyol	2, 3-Butanediol	na
Steroid	Testosterone	na
Aldehyde	Acetoin	na

The reaction mixture contained 20 mM Tris–HCl (pH 8.0), 500 µM NAD(P)^+^ and various concentrations of substrate (0.1–100 mM).

* na* no activity.

^a^NAD^+^ was used as a cofactor.

### Cofactor preference of BPSS2242

**Figure 4:**
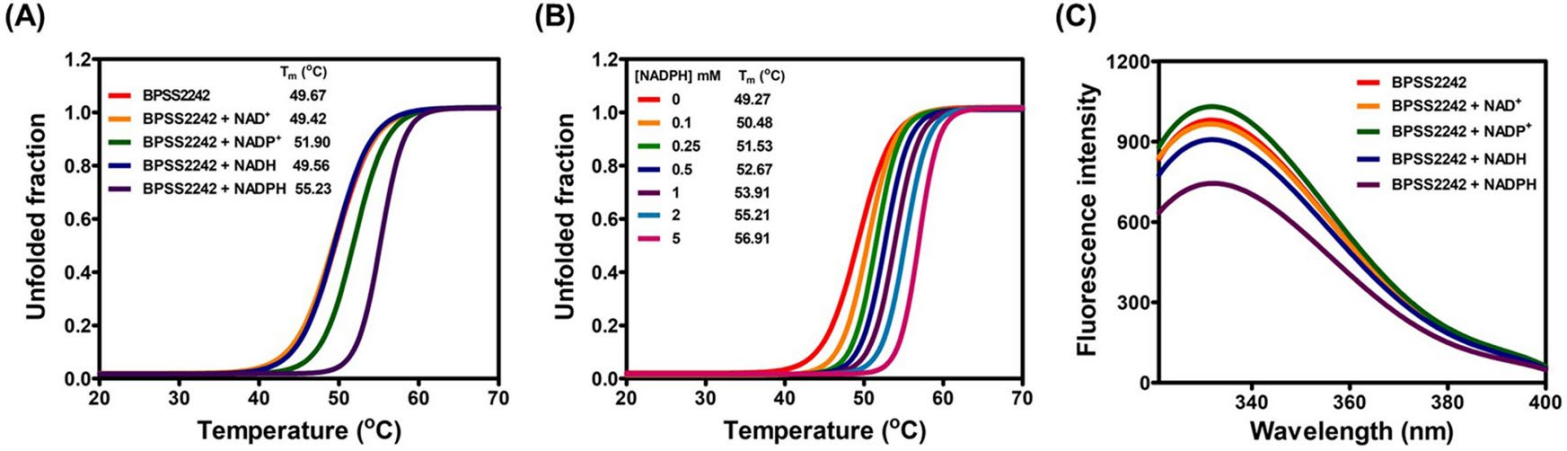
Cofactor preference of BPSS2242. (**A**) Thermal shift assays for screening cofactor preference. (**B**) Effects of NADPH concentration on thermal stability of BPSS2242. (**C**) Intrinsic fluorescence analysis for cofactor binding**.**

To assess information regarding cofactor specificity, purified BPSS2242 was subjected to cofactor binding assays. The thermal shift assay showed a significant increase in melting temperature (T_m_) in the presence of NADPH cofactor. The T_m_ value of BPSS2242 was 49.67 °C, while in the presence of 2 mM NADPH the T_m_ increased to 55.23 °C (Fig. 4A). On the other hand, no change in T_m_ was observed for BPSS2242 in the presence of NAD^+^ or NADH. In the presence of NADP^+^, T_m_ of BPSS2242 was slightly increased to 51.90 °C, which may indicate weak binding between the protein and NADP^+^. Furthermore, the effect of NADPH concentration on the thermal stability of BPSS2242 was assessed and this effect was found to be concentration-dependent (Fig. 4B).

BPSS2242 contains two tryptophan residues (Trp91 and Trp120); therefore, the structural alteration upon cofactor binding can be assessed by monitoring intrinsic tryptophan fluorescence^19^. In agreement with the conformational change suggested by the thermal shift assay, the fluorescence emission spectrum of BPSS2242 was altered in the presence of NADPH. An approximately 1.5-fold reduction in fluorescence intensity was observed for BPSS2242 in the presence of NADPH. By contrast, the presence of other cofactors slightly altered the intensity of fluorescence when compared to that of apoenzyme (Fig. 4C).

### Enzymatic properties of recombinant BPSS2242

**Figure 5:**
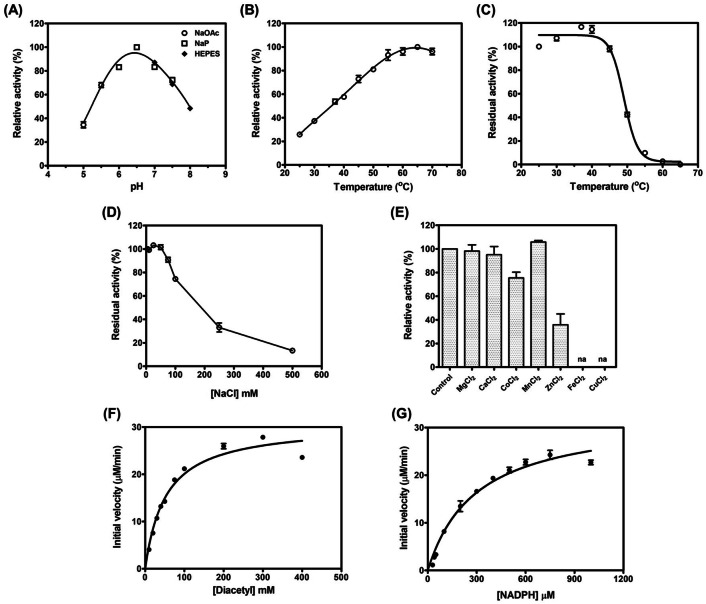
Enzymatic characterization of BPSS2242. Effects of (**A**) pH and (**B**) temperature on enzyme activity. NaOAc: sodium acetate; NaP: sodium phosphate. (**C**) Thermal stability analysis of BPSS2242. Effects of (**D**) NaCl and (**E**) metal ions on the enzyme activity. Control is the reaction without metal ion. na: no activity. Kinetic plots of BPSS2242 for (**F**) diacetyl and (**G**) NADPH.

The pH dependent activity of BPSS2242 was determined at the pH range of 5.0–8.0 and showed that the pH optimum for diacetyl reduction is 6.5 (Fig. 5A). The reaction rate increased until the temperature reached 60 °C, then decreased gradually (Fig. 5B). In thermal denaturation assay, BPSS2242 lost half of its reductase activity at 49 °C (Fig. 5C). BPSS2242 activity was inhibited in the presence of high salt concentration (higher than 75 mM) and more than half of the activity was lost when the concentration of NaCl was 250 mM (Fig. 5D). Enzyme activity was retained at NaCl concentrations between 5 and 75 mM. The addition of MgCl_2_, CaCl_2_, and MnCl_2_ had no effect on enzyme activity, while the presence of Co^2+^, Zn^2+^, Fe^2+^, and Cu^2+^ reduced the enzyme activity in which FeCl_2_ and CuCl_2_ caused severe protein precipitation (Fig. 5E).

Steady-state kinetic parameters were determined and the Michaelis–Menten plots of BPSS2242 for diacetyl and NADPH are shown in Fig. 5F and G, respectively. The *K*_m_ and *k*_*cat*_ values for diacetyl were 53 mM and 0.25 s^−1^, respectively. For NADPH, the *K*_m_ and *k*_*cat*_ values were 298 µM and 0.27 s^−1^, respectively. The catalytic efficiency of BPSS2242 toward diacetyl (0.0048 s^−1^ mM^−1^) is significantly lower than other bacterial diacetyl reductases (4.4–74 s^−1^ mM^−1^, Table 4); this suggests that diacetyl may not be the natural substrate for BPSS2242^20–29^. Though reductase activity was detected for methylglyoxal, determination of the steady-state kinetic parameters for this compound was not feasible due to the high background when the substrate concentration was increased.

**Table 4:** Kinetic constants of BPSS2242 and bacterial enzymes known to have diacetyl reductase activity.

Enzyme	Organism	Diacetyl			NADPH			References
		*K*_m_ (mM)	*k*_*cat*_ (s^−1^)	*k*_*cat*_/*K*_m_ (s^−1^ mM^−1^)	*K*_m_ (µM)	*k*_*cat*_ (s^−1^)	*k*_*cat*_/*K*_m_ (s^−1^ mM^−1^)	
BPSS2242	*B. pseudomallei*	53 ± 5	0.25 ± 0.01	0.0048 ± 0.0006	298 ± 31	0.27 ± 0.01	0.91 ± 0.11	The present study
DAR	*E. coli*	4.44	nr	nr	20	nr	nr	20
DAR	*B. polymyxa*	12	nr	nr	50	nr	nr	21
DAR^a^	*B. stearothermophilus*	19	nr	nr	nr	nr	nr	22
DAR^a^	*S. aureus*	15	nr	nr	45	nr	nr	23
DAR^a^	*E. aerogenes*	1.6	nr	nr	7	nr	nr	24
DAR^a^	*L. pseudomesenteroides*	5.1	nr	nr	nr	nr	nr	25
DAR^a^	*R. erythropolis*	0.44	1.95	4.43	770	6.56	8.52	26
BDH^a^	*C. crenatum*	0.22 ± 0.007	16.2 ± 0.9	73.64	44	68	nr	27
BDH^a^	*B. licheniformis*	72.4 ± 0.4	1,222 ± 5	16.9	250 ± 20	1,274 ± 5	5,072	28
BDH^a^	*B. clausii*	2.5 ± 0.4	48	19	nr	nr	nr	29

^a^NADH was used as a cofactor. DAR, diacetyl reductase and BDH, 2,3-butanediol dehydrogenase.

* nr* not reported.

### Roles of conserved catalytic triad on catalysis and structure of BPSS2242

**Figure 6:**
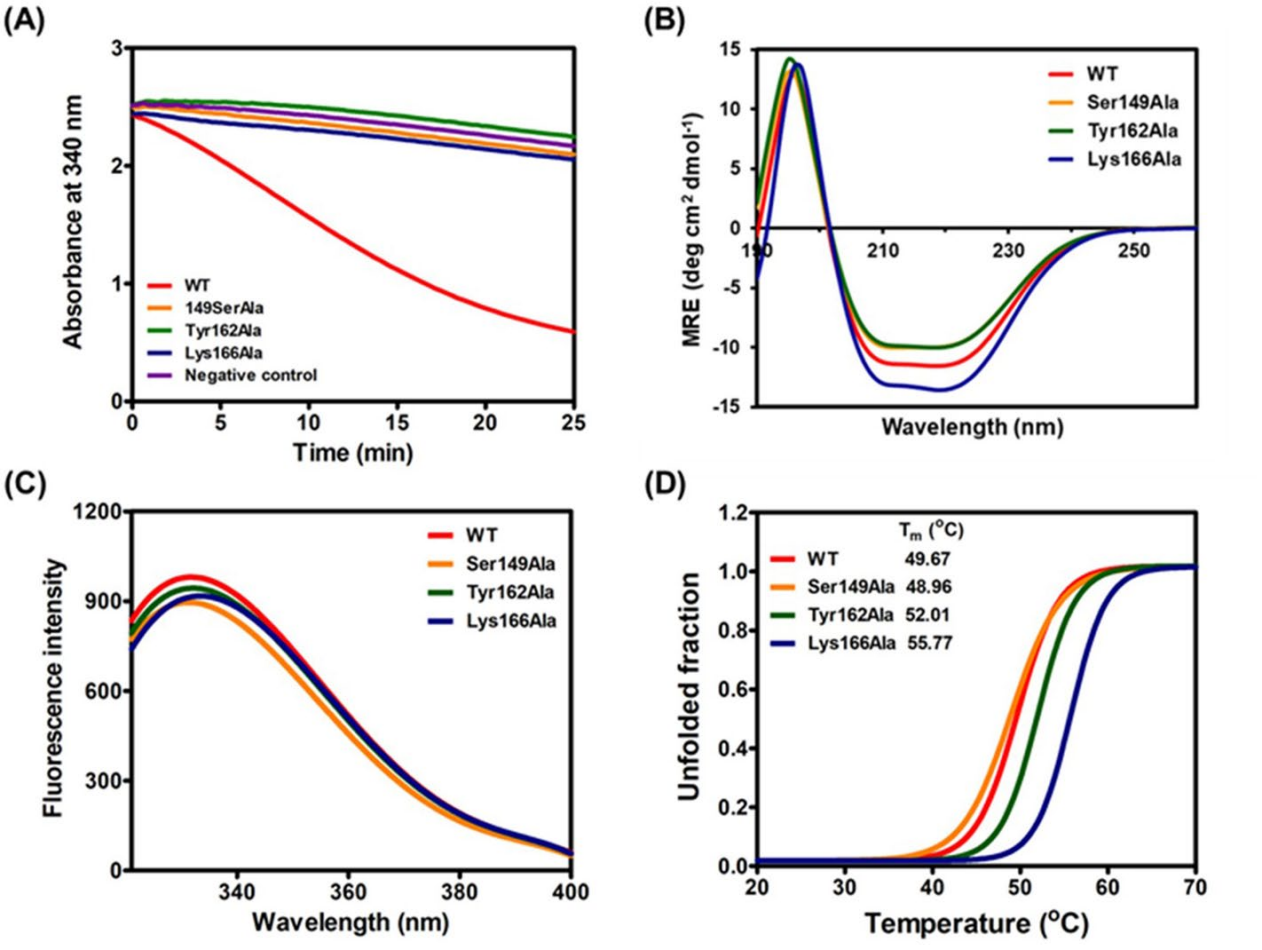
Analyses of purified BPSS2242 WT and mutants. (**A**) Comparison of diacetyl reductase activity. Negative control is the reaction without enzyme. Structural analysis by (**B**) CD and (**C**) intrinsic fluorescence. (**D**) Thermal stability analysis.

In the SDR protein superfamily, catalytic triad residues (Ser, Tyr and Lys) have been proposed as part of the catalytic site and play essential roles in enzyme catalysis^30,31^. To assess the roles of the catalytic triad of BPSS2242 in substrate specificity and catalysis, each residue was substituted with Ala and the interactions with substrate and cofactor were characterized. All mutants were catalytically inactive toward diacetyl reduction (Fig. 6A), indicating that these residues are crucial for the catalytic activity of BPSS2242.

To understand the roles of these catalytic triad residues in more detail, the effect of mutation on the secondary structure of BPSS2242 was examined (Fig. 6B). The CD spectra of Ser149Ala and Tyr162Ala were almost identical to that of the WT. Nevertheless, a decrease in intensity at 208 and 222 nm was noted. In contrary, Lys166Ala mutant showed a different CD spectrum from the WT enzyme. This mutant showed a more negative peak at 222 nm and a slight shift at 193 nm (Fig. 6B). Additionally, CDSSTR analysis demonstrated that the relative amount of α-helix was reduced in Lys166Ala mutant when compared with the WT, indicating the structural alteration caused by the mutation of catalytic Lys166 residue (Table 5).

**Table 5:** Secondary structure contents of BPSS2242, WT and mutants.

Construct	Composition of secondary structure (%)			
	α-Helix	β-Sheet	Turn	Coil
WT	60	15	13	12
Ser149Ala	61	16	12	11
Tyr162Ala	62	15	12	11
Lys166Ala	52	18	15	15

Intrinsic fluorescence analysis was also performed (Fig. 6C). Emission spectra of the Ser149Ala and Tyr162Ala mutants were similar to that of the WT enzyme with only a slight decrease in fluorescence intensity. However, the spectrum of the Lys166Ala mutant showed a shift to a longer wavelength, indicating that tryptophan residues of Lys166Ala mutant were exposed to a polar environment. This result confirmed that the mutation of Lys166 caused conformational change of BPSS2242. To further investigate the effect of mutation, the thermal stability of BPSS2242 mutants was assessed using thermal shift assay (Fig. 6D). Ser149Ala mutant had a T_m_ of 48.96 °C, comparable to the WT (T_m_ of 49.67 °C). For Tyr162Ala and Lys166Ala, the T_m_ was increased for 2.3 and 6.1 °C, respectively. The apparent change in thermal stability of the Lys166Ala mutant might result from alteration in the protein structure.

**Figure 7:**
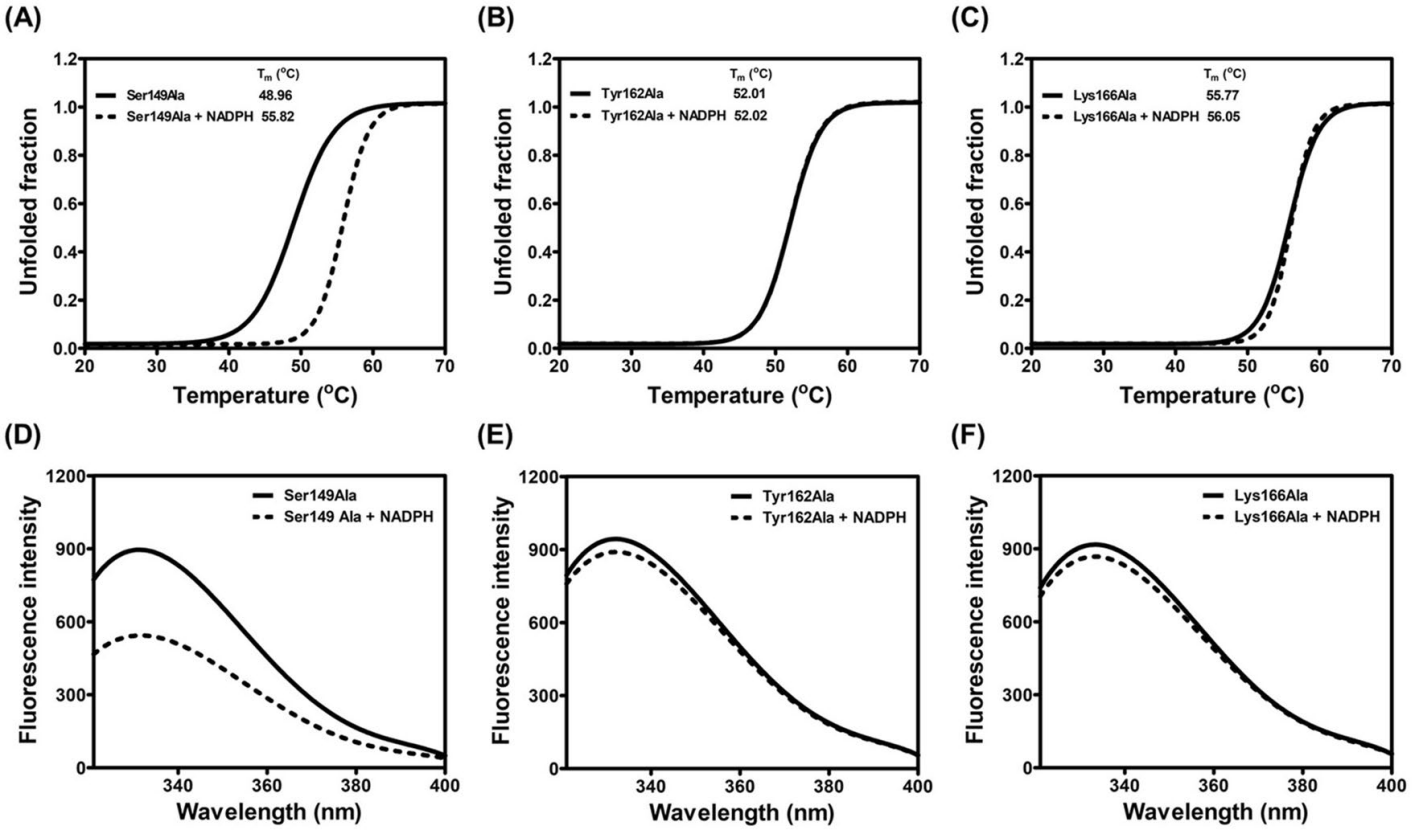
Cofactor binding analysis of BPSS2242 mutants. (**A**, **B**, and **C**) Thermal denaturation upon NADPH binding and (**E**, **F**, and **G**) the emission spectra of purified proteins in the absence and presence of NADPH.

Next, the role of the catalytic triad in cofactor binding was evaluated. In the presence of NADPH, the T_m_ of the Ser149Ala mutant was increased to 55.82 °C, in a similar manner to that observed for the WT protein (Figs. 4C, 7A). In contrast, the T_m_ of Tyr162Ala and Lys166Ala mutants in the presence of NADPH resembled that of apoenzyme, indicating no interaction between the mutant enzymes and cofactor (Fig. 7B and C). The reduction in intrinsic fluorescence emission upon binding to NADPH was only observed in the Ser149Ala mutant (Fig. 7D–F). Taken together, the study demonstrated that Tyr162 and Lys166 are involved in NADPH cofactor binding, crucial for BPSS2242 catalysis.

### The cellular function of BPSS2242 in dicarbonyl detoxification

**Figure 8:**
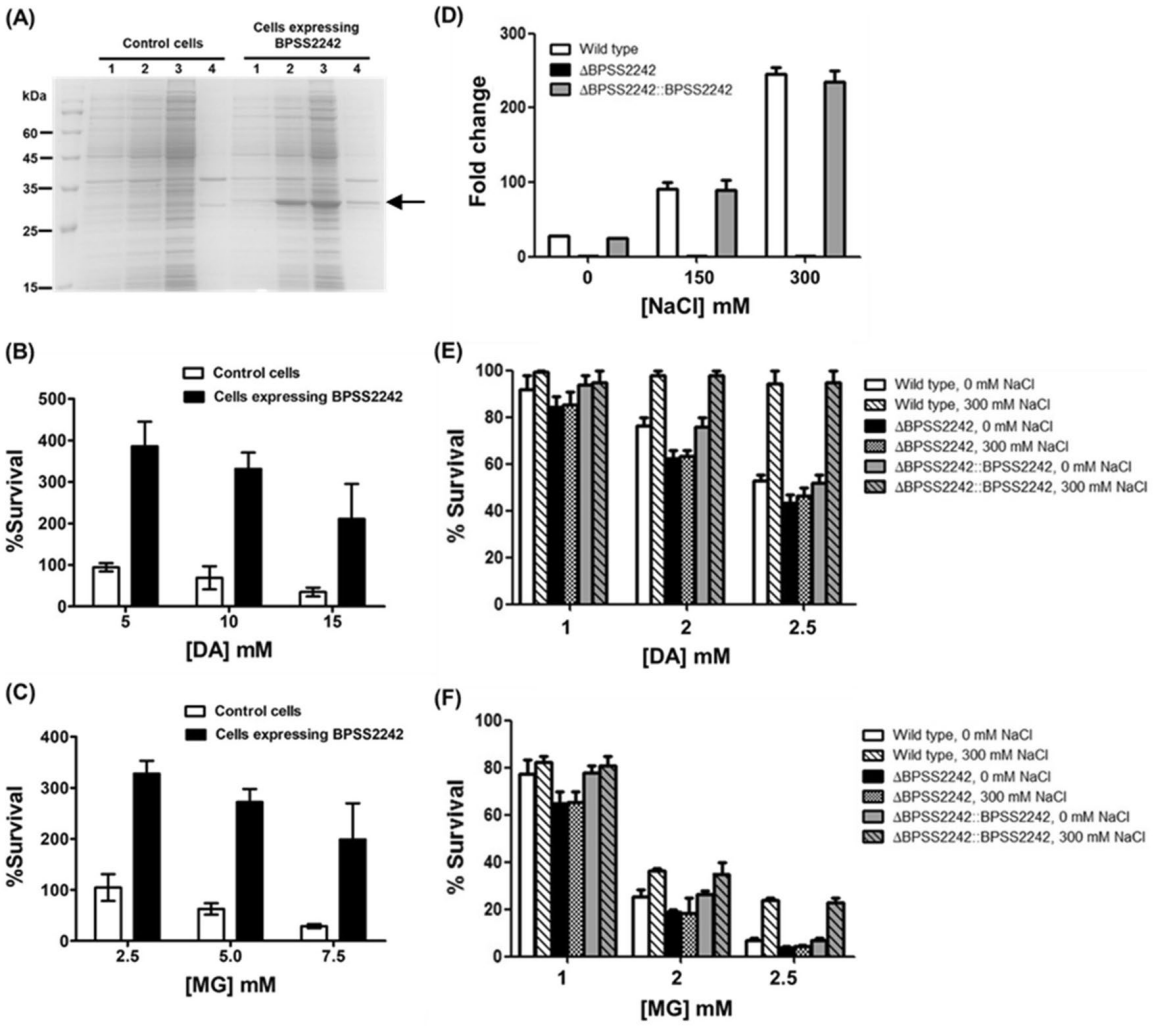
Survival of bacteria under dicarbonyl toxic stress. (**A**) Recombinant protein expression in BL21 (DE3). Lane 1, un-induced cells; lane 2, induced cells after 4 h induction with 1 mM IPTG; lane 3, soluble fraction; lane 4, insoluble fraction. Control cells are *E. coli* BL21 (DE3) carrying empty pET23a plasmid. Survival of *E. coli* was determined after exposure to (**B**) diacetyl and (**C**) methylglyoxal. (**D**) Confirmation of BPSS2242 transcript level of *B. pseudomallei* grown in LB medium containing 0, 150 mM and 300 mM NaCl by RT-PCR. The survivals of salt-treated and untreated *B. pseudomallei* WT, △BPSS2242 mutant, and complemented strains after exposure to (**E**) diacetyl and (**F**) methylglyoxal.

Since BPSS2242 is able to catalyze the reduction of diacetyl and methylglyoxal that are known as reactive electrophilic species, damaging macromolecules and affecting cellular redox status^32,33^, we hypothesized that overexpression of BPSS2242 could provide protection against dicarbonyl toxicity. Therefore, the biological role of BPSS2242 was assessed in both *E. coli* and *B. pseudomallei*. For *E. coli* BL21 (DE3), bacterial survival of cells expressing BPSS2242 was compared with that of bacteria harboring empty pET23a plasmid (control). After 4 h of BPSS2242 overexpression in *E. coli* induced by IPTG (Fig. 8A), the bacteria were exposed to various concentrations of diacetyl (0–15 mM) and methylglyoxal (0–7.5 mM) for 1 h and the surviving cell numbers were counted to evaluate the toxicity effect of dicarbonyl compounds (Fig. 8B and C). In the presence of dicarbonyl compounds, the survival of *E. coli* BL21 (DE3) expressing BPSS2242 was significantly greater than that of the control at all concentrations tested. This indicated that the expression of BPSS2242 provides some advantages for the growth/survival of bacteria. With increasing concentrations of dicarbonyls, bacterial survival decreased in a concentration-dependent manner where the survival of control cells was more severely reduced than that of BPSS2242 overexpressing cells.

To examine the detoxifying function of BPSS2242 in *B. pseudomallei*, the previously constructed △BPSS2242 mutant^14^ was used. The survival of WT, △BPSS2242 and BPSS2242 complemented *B. pseudomallei* grown in media supplemented with different concentrations of NaCl and dicarbonyl compounds was determined. Initially, RT-PCR was carried out to assess the expression of BPSS2242. The results showed that the expression of BPSS2242 in WT *B. pseudomallei* grown in the presence of 300 mM NaCl was up-regulated (Fig. 8D), in good agreement with previous finding^11^. The △BPSS2242 mutant did not show BPSS2242 expression while the expression of BPSS2242 in the complemented strain was comparable to that observed in WT. After exposure to 2.5 mM diacetyl, the survival of △BPSS2242 *B. pseudomallei* was radically reduced to 40–50%, in contrast with that of the WT in which the survival was still more than 90% (Fig. 8E). Additionally, the survival of △BPSS2242 mutant was retrieved to be similar to the WT when it was complemented with BPSS2242. It was clearly demonstrated that increasing NaCl concentration from 0 to 300 mM enhanced the survival of *B. pseudomallei* in WT and complemented strains which is correlated with increased BPSS2242 expression induced by NaCl. For methylglyoxal toxicity, the survival of WT *B. pseudomallei* was significantly improved and BPSS2242 complemented strain recovered the survival of △BPSS2242 in manner similar to the WT in the presence of 300 mM NaCl (Fig. 8F). These results provide evidence that BPSS2242 is capable of detoxifying dicarbonyl compounds (diacetyl and methylglyoxal) in *B. pseudomallei*.

## Discussion

*B. pseudomallei* is a versatile saprophyte that can survive under different types of stress^3,4^. The persistence of *B. pseudomallei* in extreme environments probably contributes to the risk of infection in endemic areas. A previous study showed the up-regulation of *bpss2242* in *B. pseudomallei* cultured under high NaCl concentration^11^. In addition, invasion and early intracellular replication of BPSS2242 *B. pseudomallei* were impaired, suggesting its important role in survival and pathogenesis^14^. BPSS2242 was identified as a putative SDR; however, its biochemical and functional roles are still unknown.

Bioinformatics analysis revealed a unique gene structure of *bpss2242* in *B. pseudomallei* isolate K96243, a reference strain, used in this study (Supplementary Fig. S4). Unlike BPSS2242 of K96243, other *B. pseudomallei* isolates contain additional part which was identified as BPSS2241 in K96243, indicating that BPSS2242 and BPSS2241 are fused into a single protein in other *B. pseudomallei* isolates.

Upon substrate screening for BPSS2242, BPSS2242 mixed with BPSS2241 and BPSS2242 + 41, no enzymatic activity was observed for BPSS2241 against all substrates tested. BPSS2242 + 41 showed enzymatic activity comparable to BPSS2242. In contrast to BPSS2242, BPSS2241 was not up-regulated when *B. pseudomallei* K96243 was cultured in NaCl-supplemented media^11^, suggesting that BPSS2241 is not implicated in catalysis. This is in good agreement with the fact that no conserved residue of SDR was found on BPSS2241. BPSS2241 is a small protein of 69 amino acids with an expected molecular mass of 7.4 kDa. Additionally, a sequence query for BPSS2241 in the non-redundant protein database could not identify a potential candidate. BPSS2241 was predicted using TMHMM to have transmembrane (TM) helix characteristics, suggesting its function as a membrane-anchoring protein^34^. CD analysis also indicated that the α-helix content of BPSS2241 is higher than that of BPSS2242 and BPSS2242 + 41. Some SDRs were reported to contain additional N- or C-terminal transmembrane region or signal peptides extended from the core structure^30^. BPSS2241 probably plays role in stabilizing BPSS2242 + 41, since the degradation of BPSS2242 + 41 was diminished during purification (Supplementary Fig. S3). On the other hand, the unique structural organization of *bpss2242* observed in *B. pseudomallei* isolate K96243 might be explained by the genetic diversity of environmental *B. pseudomallei* K96243 samples^35–37^.

A previous study reported GDH activity detected in crude lysate of *B. pseudomallei* K96243 grown in the presence of 300 mM NaCl as a consequence of BPSS2242 expression^14^. Nonetheless, BPSS2242 showed no GDH activity. Both BPSS2242 and BPSS2242 + 41 exhibited reductase activity toward dicarbonyl compounds, diacetyl and methylglyoxal and the activity was dependent on NADPH. BPSS2242 was suggested as an NADP(H)-preferring classical SDR, in which a basic residue (Arg42) is present in the loop after the second β-strand. This basic residue was proposed to interact with the 2′-phosphate of NADP(H)^13,38,39^.

The preference for NADPH coenzyme of BPSS2242 was confirmed by thermal shift and intrinsic fluorescence analyses. The T_m_ of BPSS2242 was significantly increased from 49.67 °C to 55.23 °C in the presence of 2 mM NADPH. Furthermore, a change in intrinsic fluorescence signal was only observed in the presence of NADPH, indicating the conformational change upon NADPH binding. Though a shift in T_m_ of 2.23 °C was noted in the presence of NADP^+^, no dehydrogenase activity was detected toward any substrate tested. In this context, it was demonstrated that BPSS2242 is an NADPH-dependent reductase.

Similar to other SDRs, the proposed catalytic triad–Ser149, Tyr162 and Lys166–in BPSS2242 was found to be critical for catalysis^30,31^. Site-directed mutagenesis of these residues to Ala abolished enzyme activity. The Tyr162Ala and Lys166Ala mutants showed the T_m_ of 52.01 °C and 55.77 °C, respectively while Ser149Ala shared similar T_m_ (48.96 °C) to the WT protein. As suggested by CD and intrinsic fluorescence analyses, Lys166Ala altered the secondary structure where the α–helix content was decreased and this mutant also caused a conformational change to the protein. Tyr was proposed to act as a catalytic acid/base, transferring the proton from NADPH to the carbonyl group of the substrate while Lys was found to bind to NADPH and lower pK_a_ of Tyr to facilitate proton transfer^30,31^. In this study, thermal shift and intrinsic fluorescence analyses demonstrated that Tyr162Ala and Lys166Ala are involved in NADPH binding. Mutation of these two catalytic residues disrupted protein binding to NADPH coenzyme.

It should be mentioned that while BPSS2242 can catalyze the reduction of diacetyl, the *K*_m_ for diacetyl (53 mM) and NADPH (298 µM) are higher than those reported for other SDR members, except those of *B. licheniformis* diacetyl reductase for which the values are comparable (*K*_m_ of 72.4 mM and 250 µM for diacetyl and NADPH, respectively) (Table 4)^28^. However, *B. licheniformis* diacetyl reductase is a highly catalytic efficient enzyme (*k*_*cat*_/*K*_m_ of 16.9 s^−1^ mM^−1^ and 5,027 s^−1^ mM^−1^ for diacetyl and NADPH, respectively). The low binding affinity and catalytic efficiency of BPSS2242 toward diacetyl reduction may suggest that diacetyl compound is not the natural substrate of this enzyme. BPSS2242 showed two major distinct characteristics from other diacetyl reductases. First, diacetyl reductase activity of BPSS2242 is irreversible i.e. unable to catalyze the oxidation of acetoin (Table 3). However, irreversible diacetyl reduction was reported for some diacetyl reductase enzymes^22,25^. Second, BPSS2242 activity is dependent on NADPH while most of bacterial diacetyl reductases utilize NADH as a cofactor^22–26^. Diacetyl reductase activity of BPSS2242 shared similar features to *E. coli* diacetyl reductase where it preferred NADPH as a cofactor and was unable to oxidize acetoin substrate. However, the *K*_m_ values of *E. coli* diacetyl reductase for NADPH and diacetyl are 20 µM and 4.44 mM, respectively, ten-fold lower than those observed for BPSS2242^20^.

Diacetyl and methylglyoxal are dicarbonyl compounds recognized for their oxidative toxicity in many organisms^32,33^. They were reported to cause a loss in cell viability and to inhibit growth; hence, they have been utilized as antimicrobials^40,41^. Methylglyoxal is endogenously synthesized from hydroxyacetone phosphate by glycerol metabolism, glucose oxidation, lipid peroxidation and DNA oxidation in various organisms, including *E. coli* and *B. pseudomallei*^42–45^. On the other hand, diacetyl or 2, 3-butanedione is an α–diketone naturally synthesized during fermentation^46^. The NAD(P)H-dependent enzymes of SDR superfamily and the aldo–keto reductase (AKR) superfamily are two major enzymatic pathways responsible for detoxification of dicarbonyl compounds^33,47–49^. NADPH-dependent aldehyde reductases (ADRs) together with AKR and alkenal/one oxidoreductase cooperatively scavenge dicarbonyl in plant *Arabidopsis*^49^. *Arabidopsis* ADRs could reduce saturated aldehydes such as propionaldehyde and butyraldehyde in the presence of NADPH, but not NADH. Similar to BPSS2242, the ADR reaction is irreversible at physiological pH, suggesting the physiological significance of ADRs in elimination of aldehydes. Furthermore, co-expression of ADR, AKRs and alkenal reductase in *Arabidopsis* was reported to function together to detoxify aldehydes produced under conditions of stress, such as high salinity and drought. Likewise, *E. coli* ADR could eliminate aldehydes produced during lipid peroxidation^50^. In this study, we have shown that overexpression of BPSS2242, a putative SDR, significantly increased *E. coli* and *B. pseudomallei* K96243 survival when bacteria were exposed to diacetyl and methylglyoxal at their physiological concentrations^41,42,51^. This demonstrated the ability of BPSS2242 to detoxify diacetyl and methylglyoxal in vivo.

Generally, microorganisms possess their survival adaptability under stress conditions. *B. pseudomallei* K96243 was shown to tolerate a wide range of stress conditions, including high salt condition^4,8,9^. Metabolic changes were observed when *B. pseudomallei* K96243 encountered 300 mM NaCl, such as up-regulation of enzymes in sugar metabolisms, pyruvate dehydrogenase and UDP-galactose 4-epimerase^10^. The increase of glycolytic activity may provide energy required for survival^10^. The bacteria might generate cytotoxic dicarbonyl compounds as a consequence of increased glycolytic activity and lipid metabolisms. From our results, it is suggested that up-regulation of BPSS2242 observed in *B. pseudomallei* K96243 grown under high salt concentration might be one of the mechanisms or partially responsible for reducing the oxidative toxicity of dicarbonyl compounds, protecting the bacteria under salt stress.

## Materials and methods

### Materials

Phusion High Fidelity DNA polymerase was obtained from Thermo Scientific (Waltham, MA, USA). T4 ligase and restriction enzymes were purchased from New England Biolabs (Ipswich, MA, USA). All bacteria media were supplied by Bio Basic (Markham, ON, Canada). NAD(P)^+^, NAD(P)H and sugars were purchased from Sigma-Aldrich (St. Louis, MO, USA). Alcohols, ketones, aldehydes and other chemicals were purchased from Merck (Darmstadt, Germany) and TCI (Tokyo, Japan). All chemicals used were of analytical grade and were used as received.

### Secondary structure analysis by CD

The secondary structure of purified proteins was assessed using CD spectroscopy. NaCl was removed by buffer exchange against 20 mM Tris–HCl pH 8.0 containing 10% glycerol using Amicon Ultra centrifugal filter device (Merck Millipore, MA, USA). Far UV-CD spectra of the BPSS2242, BPSS2241, and BPSS2242 + 41 at a protein concentration of 0.2 mg/mL were recorded using a Jasco spectrometer, model J-815, with a 1 mm path length quartz cuvette. The spectra were collected over a wavelength range of 190–260 nm at a scan rate of 50 nm/min. Five scans were averaged for each sample and subtracted with the buffer scan.

### Determination of molecular mass

The native molecular mass of the purified BPSS2242 was determined by size exclusion chromatography using AKTA fast protein liquid chromatography (FPLC) equipped with Superdex 200 Increase 10/300 column (GE Healthcare, NJ, USA). The purified protein (50 µg) was loaded onto pre-equilibrated column. The chromatography was performed at a flow rate of 0.5 mL/min using 20 mM Tris–HCl pH 8.0 containing 500 mM NaCl. The column was calibrated with blue dextran (> 2000 kDa), ferritin (440 kDa), catalase (232 kDa), aldolase (158 kDa), ovalbumin (43 kDa), chymotrypsinogen (25 kDa), and RNase A (13.7 kDa). Calibration curve was constructed by plotting the distribution coefficient (K_av_) versus the logarithm of protein molecular weight using the equation: K_av_ = (V_e_/V_o_)/(V_c_ -V_o_), where V_c_ is the total bed volume, V_o_ is the void volume, and V_e_ is the elution volume.

### Thermal shift assay

To identity cofactor that could bind to BPSS2242, thermal shift assay based on fluorimetry was performed in 20 µL reaction mixture containing 3 µM BPSS2242 mixed with 5 × SYPRO Orange reporter dye and 2 mM nucleotide cofactors (NAD^+^, NADP^+^, NADH, and NADPH) in 20 mM Tris–HCl pH 8.0. The reaction mixtures were heated in an increment of temperature ranging from 20 to 70 °C using LightCycler 480 real-time PCR machine (Roche, Mannheim, Germany) with excitation and emission wavelengths of 465 nm and 580 nm, respectively. T_m_ of each BPSS2242-nucleotide pair was determined. The T_m_ was calculated by a Boltzmann fit using GraphPad Prism. A shift in T_m_ larger than 2 °C was considered to be statistically significant.

### Intrinsic fluorescence analysis

Conformational change of recombinant BPSS2242 upon cofactor binding was investigated by intrinsic tryptophan fluorescence. A total volume of 100 µL contained 2.5 µM BPSS2242 in the absence or presence of 25 µM of either NAD^+^, NADP^+^, NADH, or NADPH. Intrinsic fluorescence spectra of recombinant BPSS2242 and holoenzyme were collected using Synergy H1 Hybrid Reader (BioTek, VT, USA) in a 96-well plate at 25 °C. The excitation wavelength was 295 nm and the emission spectra were recorded in the range of 300–400 nm.

### Enzyme assay and determination of kinetic parameters

Enzyme activity was measured spectrophotometrically by monitoring the change in absorbance of NAD(P)H at 340 nm, using a molar extinction coefficient of 6,220 M^−1^ cm^−1^. Recordings were carried out with UV-2700 UV–VIS spectrophotometer (Shimadzu, Kyoto, Japan). The standard reaction contained 20 mM sodium phosphate (pH 6.5), 500 µM NADPH, 200 mM diacetyl and 50 mM NaCl and was performed at 37 °C in a cuvette with a final volume of 1 mL.

Purified recombinant SDRs were subjected to activity assay towards different substrates, including sugars, alcohols, polyol, steroid, ketones, aldehydes, and fatty acid using NAD(P)(H) as cofactors.

To determine kinetic parameters, diacetyl and NADPH were used as substrates. For the determination of *K*_m_ for NADPH, the assay was performed by fixing the concentration of diacetyl at 200 mM and varying the concentrations of NADPH from 30–1,000 µM, while the *K*_m_ for diacetyl was determined by fixing the concentration of NADPH at 500 µM and varying the concentrations of diacetyl from 10–400 mM. The kinetic constants were calculated by fitting the initial velocity to Michaelis–Menten equation using GraphPad Prism.

### Effect of pH on enzyme activity

To study the effect of pH, the enzyme activity was measured in the presence of 20 mM of each of the following buffers: sodium acetate (pH 5.0–5.5), sodium phosphate (pH 6.0–7.5), and HEPES (pH 7.0–8.0).

### Effect of temperature on enzyme activity and thermal stability assay

To investigate the effect of temperature, the enzyme assay was carried out at the temperatures ranging from 25 to 70 °C. For thermal stability analysis, the purified enzyme was pre-incubated at different temperatures ranging from 25 to 65 °C for 20 min, and was then cooled down to 4 °C in a Thermocycler (Eppendorf, Hamburg, Germany). Residual activity of the enzyme was determined under standard condition and expressed as a percentage of the activity of the enzyme incubated at 25 °C.

### Effects of metal ions and salt on enzyme activity

The effect of metal ions on BPSS2242 activity was investigated in the standard reaction mixture containing 1 mM of different divalent metal ions (Mg^2+^, Ca^2+^, Co^2+^, Mn^2+^, Zn^2+^, Fe^2+^, and Cu^2+^). The effect of NaCl itself on BPSS2242 activity was determined by measuring enzyme activity in the presence of different concentrations of NaCl (5–500 mM).

### Effect of dicarbonyl compounds on survival of bacteria expressing BPSS2242

*E. coli* BL21 (DE3) cells harboring pET23a-*bpss2242* or empty pET23a plasmid were grown in Luria–Bertani (LB) at 37 °C until OD_600_ reached 1.0 and protein expression was induced by addition of 1 mM IPTG and further cultured at 20 °C for 4 h. Thereafter, cells were treated with various concentrations of diacetyl (5–15 mM) or methylglyoxal (2.5–7.5 mM) for 1 h. The concentrations of diacetyl and methylglyoxal used in this study were chosen based on previous reports^41,48,52,53^. For viable cell counts, cultures were serially diluted ten-fold in LB broth and plated onto LB agar containing 100 µg/mL ampicillin.

The survival of *B. pseudomallei* K96243 after exposure to dicarbonyl compounds was determined. In this study, we compared the survival of three strains of *B. pseudomallei* K96243: WT, △BPSS2242 mutant, and the *bpss2242* complemented strain. Construction of the △BPSS2242 mutant and *bpss2242* complemented strains has been described previously^14^. The expression of BPSS2242 was induced by growing *B. pseudomallei* in 0, 150 and 300 mM of NaCl and validated by real-time RT-PCR as previously described^54^. In brief, *B. pseudomallei* were cultured in the presence or absence of NaCl at 37 °C for 6 h and RNA isolation was performed by adding 10 mL of RNAprotect Bacterial Reagent (QIAGEN, TX, USA) to 5 mL of bacterial culture and incubating for 5 min at room temperature. Thereafter, total RNA was extracted from bacterial pellets using TRIzol (Invitrogen, CA, USA) according to the manufacturer's instructions. To remove the DNA, the solution was treated with DNase (NEB, MA, USA) for 10 min at 37 °C before use. Conventional PCR for 23S RNA gene was performed to verify that there was no contamination of gDNA in the DNase-treated RNA samples. Subsequently, real time RT-PCR was carried out for the *bpss2242* gene using KAPA SYBR fast one-step (Kapa Biosystems, MA, USA) with following conditions: reverse transcription at 50 °C for 30 min, enzyme activation at 95 °C for 10 min, then 40 cycles of denaturation at 95 °C for 30 s, annealing at 55 °C for 1 min, and melting curve analysis at 72 °C for 1 min in a CFX96 Touch Real-Time PCR Detection System (Bio-Rad, CA, USA). Real-time RT-PCR primers for BPSS2242 expression are BPSS2242-F1 (5′ ACCGCGCGACCGATATGAACG 3′) and BPSS2242-R2 (5′ TCCCTTCGCGCTCGTGCAAC 3′). Relative mRNA levels were determined by fold-change in expression, calculated by 2^− ΔΔCT^ using the relative mRNA level of 23S RNA, representing a house-keeping gene expression, as a baseline for comparison.

To evaluate the effect of dicarbonyl compounds on the survival of *B. pseudomallei* K96243 expressing BPSS2242, overnight cultures of *B. pseudomallei* K96243 (WT, deleted mutant and complementary stains) adjusted OD_600_ to 0.5 was inoculated into LB broth containing 0, 150 and 300 mM NaCl and cultured at 37 °C for 6 h. Both salt-treated and untreated bacteria were incubated with various concentrations of diacetyl (1–15 mM) or methylglyoxal (1–7.5 mM). However, both diacetyl and methylglyoxal are highly toxic to *B. pseudomallei*. Hence, the concentrations used for determination of cell viability are 1–2.5 mM for both compounds. After 1 h, cells were serially diluted ten-fold and plated for colony count. %Survival = CFU (with toxic dicarbonyl compound) × 100/CFU (without toxic dicarbonyl compound).

## Supplementary information

Supplementary information.

## Data Availability

All data generated or analyzed during this study are included in this published article and its supplementary information.
