# Supplementary material for: Functional analysis of BPSS2242 reveals its detoxification role in Burkholderia pseudomallei under salt stress
Source: Sci Rep. 2020 Jun 26;10:10453. doi: 10.1038/s41598-020-67382-y (PMC7320009; doi:10.1038/s41598-020-67382-y)
Supplement: Supplementary file 1 — Supplementary information. [file 41598_2020_67382_MOESM1_ESM.docx]

Supplementary information

Functional analysis of BPSS2242 reveals its detoxification role in *Burkholderia pseudomallei* under salt stress

Kamonwan Chamchoy^1^, Pornpan Pumirat^2^, Onrapak Reamtong^1^,

Danaya Pakotiprapha^3^, Ubolsree Leartsakulpanich^4^, and Usa Boonyuen^1,^*

^1^ Department of Molecular Tropical Medicine and Genetics, Faculty of Tropical Medicine, Mahidol University, Bangkok, 10400, Thailand

^2^ Department of Microbiology and Immunology, Faculty of Tropical Medicine, Mahidol University, Bangkok, 10400, Thailand

^3^ Department of Biochemistry, Faculty of Science, Mahidol University, Bangkok, 10400, Thailand; Center for Excellence in Protein and Enzyme Technology, Faculty of Science, Mahidol University, Bangkok, 10400, Thailand

^4^ National Center for Genetic Engineering and Biotechnology, National Science and Technology Development Agency, Pathumthani, 12120, Thailand

***** email: usa.boo@mahidol.ac.th

**Experimental**

**Cloning of *bpss2242*, *bpss2241* and *bpss2242+41* and site-directed mutagenesis**

The full-length *bpss2242* was amplified from genomic DNA of *B. pseudomallei* K96243. Two restriction sites, *BamH*I and *Hind*III, were introduced in the forward and reverse primers, respectively. The amplified products were gel purified and digested. Subsequently, the digested amplicon was cloned into pET23a expression vector. A recombinant pET28a plasmid containing *bpss2241*, a locus downstream of *bpss2242*, was also constructed. Additionally, a recombinant pET28a containing both *bpss2242* and *bpss2241* as a single gene (*bpss2242+41*) was synthesized by Gen script (Piscataway, NJ, USA).

The predicted catalytic residues of BPSS2242–Ser149, Tyr162, and Lys166–were identified and mutated to Ala by site-directed mutagenesis using pET23a-*bpss2242* WT as a template. The polymerase chain reaction (PCR) mixture (50 µL) comprised 1x HF Phusion buffer, 200 μM of each dNTP, 1 U of Phusion HF DNA polymerase, 100 ng of template plasmid, and 0.5 μM of primers. The cycling parameters for site-directed mutagenesis are as follows: 1 cycle of 98 ^o^C for 30 sec, 25 cycles of 98 ^o^C for 10 sec, 60 ^o^C for 30 sec, and 72 ^o^C for 2 min and 30 sec. PCR products were digested with *Dpn*I at 37 ^o^C for 2 hours to specifically digest the DNA templates, followed by transformation into *E. coli* DH5α competent cells. All constructs were verified by restriction digestion and DNA sequencing. The primers used for gene cloning and site-directed mutagenesis are in Supplementary Table S1.

| **Table** **S1.** List of primers used in gene cloning and site-directed mutagenesis. | |
| --- | --- |
| Primer | Sequence |
| BPSS2242_F | 5’ CCGGGATCCATGAAATGCACGCTGAAG 3’ |
| BPSS2242_R | 5’GTGAAGCTTCCCGCGCGAGAACAGCGACGTGGCGAGCCG 3’ |
| BPSS2241_F | 5’ CTAGGATCCATGCGCCCCGCGCGGCCGCGCGACGAC 3’ |
| BPSS2241_R | 5’ GTGAAGCTTCTACGAAGCGACGCCGCGCCGAGC 3’ |
| Ser149Ala_F | 5’ ATCAACATGGGCGCCGAGGCGTCC 3’ |
| Ser149Ala_R | 5’ GGACGCCTCGGCGCCCATGTTGAT 3’ |
| Tyr162Ala_F | 5’ CTGCAAAGCGCGGCTGCGGCATCC 3’ |
| Tyr162Ala_R | 5’ GGATGCCGCAGCCGCGCTTTGCAG 3’ |
| Lys166Ala_F | 5’AGCGCGTATGCGGCATCCGCGCACGCG 3’ |
| Lys166Ala_R | 5’ CGCGTGCGCGGATGCCGCATACGCGCT 3’ |

**Protein overexpression and purification**

A single colony of BL21 (DE3) harboring the desired plasmid was inoculated in LB media containing 100 µg/mL ampicillin for pET23a-*bpss2242* or 50 µg/mL kanamycin for pET28a-*bpss2241* and pET28a-*bpss2242+41* and incubated at 37 ^o^C with 250 rpm shaking overnight. Fresh overnight cultures were inoculated in LB media at a dilution of 1:100 and grown at 37 ^o^C with 250 rpm shaking until OD_600_ reached 0.8-1. Then, the protein expressions were induced with IPTG at a final concentration of 1 mM, followed by additional cultured for 20 hours before harvesting by centrifugation at 3,000x*g* for 15 minutes. Expression of BPSS2242 and BPSS2242+41 was induced at 20 ^o^C while that of BPSS2241 was done at 37 ^o^C in the presence of 10% glycerol.

For protein purification of BPSS2242, cell pellets were resuspended in lysis buffer (20 mM sodium phosphate pH 7.4, 500 mM NaCl and 10 mM imidazole), disrupted by sonication and subjected to centrifugation at 20,000x*g* for 1 hour. The supernatant was collected and incubated with TALON metal affinity resin at 4 ^o^C for 1 hour. The unbound proteins were removed using wash buffer (20 mM sodium phosphate pH 7.4, 500 mM NaCl and 20 mM imidazole). The protein was eluted with increasing imidazole concentrations from 40 to 400 mM in lysis buffer. Each eluted fraction was subjected to SDS-PAGE to determine the protein purity. Then, the fractions containing BPSS2242 were pooled and overnight dialyzed against 20 mM Tris-HCl pH 8.0 in the presence of 500 mM NaCl and 10% glycerol. Finally, the protein concentration was determined by the Bradford assay.

BPSS2241 and BPSS2242+41 proteins were purified using protocol mentioned above with some modifications. All buffers used for these protein purifications did not contain salt and purified proteins were dialyzed against 20 mM Tris-HCl pH 8.0 containing 10% glycerol. The expression of recombinant proteins was verified by Western blotting using anti-His antibody.

**Results**

**Sequence analysis reveals a unique character of BPSS2242**

Alignment of *bpss2242* and *sdr* from other *Burkholderia spp*. revealed high sequence identity among the pathogenic *Burkholderia*. Surprisingly, sequences that show high identity with *bpss2242* contain additional sequence at the 3’-end, which is not found in *bpss2242* isolate K96243 used in this study (Supplementary Fig. S1). This additional sequence was identified as *bpss2241* (NCBI Reference Sequence: YP_112244.1) which is the locus downstream of *bpss2242*.

**Cloning of bpss2242, bpss2241 and bpss2242+41**

The *bpss2242* gene from *B. pseudomallei* K96243 is 816 bp in length. The downstream gene, *bpss2241*, is 210 bp in size and coded for a 69-amino acid protein. Amplification of *bpss2242+41* was also attempted (Supplementary Fig. S2). DNA sequencing of *bpss2242+41* PCR products revealed a stop codon between *bpss2242* and *bpss2241*, indicating the separation of *bpss2242* and *bpss2241* into two loci in *B. pseudomallei* K96243. This is a unique characteristic that differentiates K96243 from other *B. pseudomallei* pathogenic isolates.

**Purification of the recombinant BpSDRs**

During purification, the recombinant BPSS2242 was unstable i.e. easily precipitated. SDS-PAGE analysis of purified protein showed two bands near the expected size of 30 kDa (Supplementary Fig. S3A). The presence of 500 mM NaCl improved protein stability and the protease cleavage was prevented by addition of phenanthroline, an inhibitor of metalloproteases. In contrast to BPSS2242, BPSS2242+41 and BPSS2241 did not degrade during purification. However, they were precipitated in the presence of NaCl. Therefore, the purification of these two proteins was carried out in the absence of NaCl. SDS-PAGE and Western blotting of purified recombinant BPSS2242, BPSS2242+41, and BPSS2241 are shown in Supplementary Fig. S3B and S3C, respectively.

**
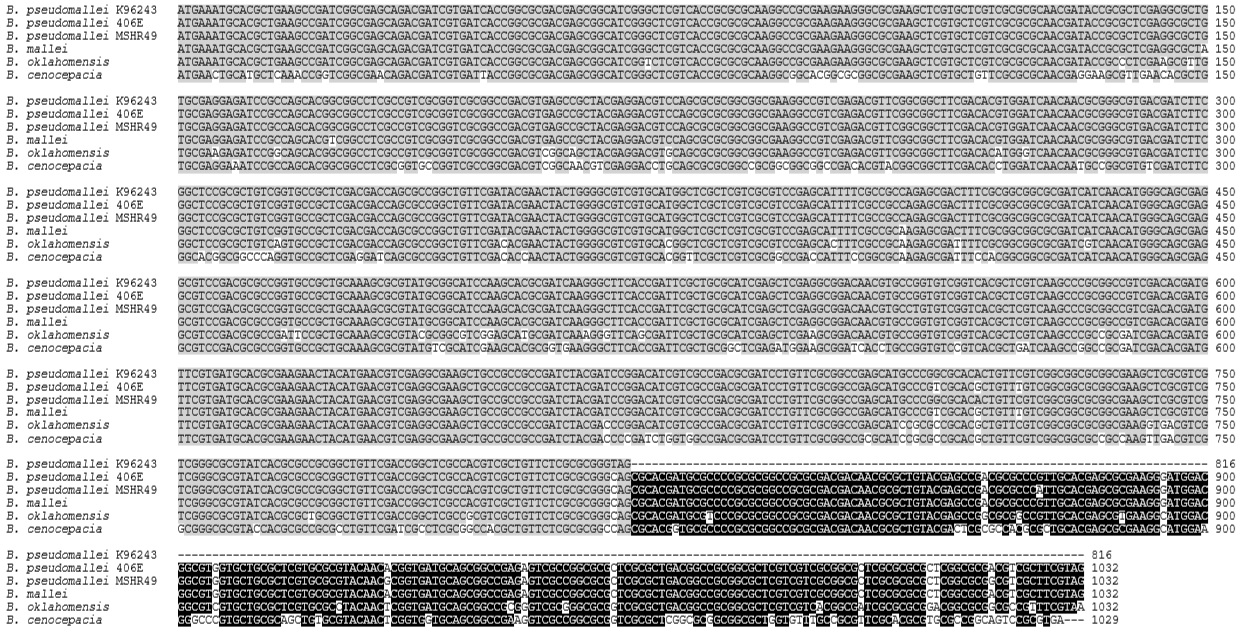
Figure S1.** Multiple sequence alignment of *bpss2242* from *B. pseudomallei* K96243 and *sdr* from pathogenic *Burkholderia*. Among other *Burkholderia* isolates, there are additional nucleotides added to 3’-end that is absent in *bpss2422*. Homologs of *bpss2242* are shaded in gray. Additional sequence was identified as *bpss2241* (black).


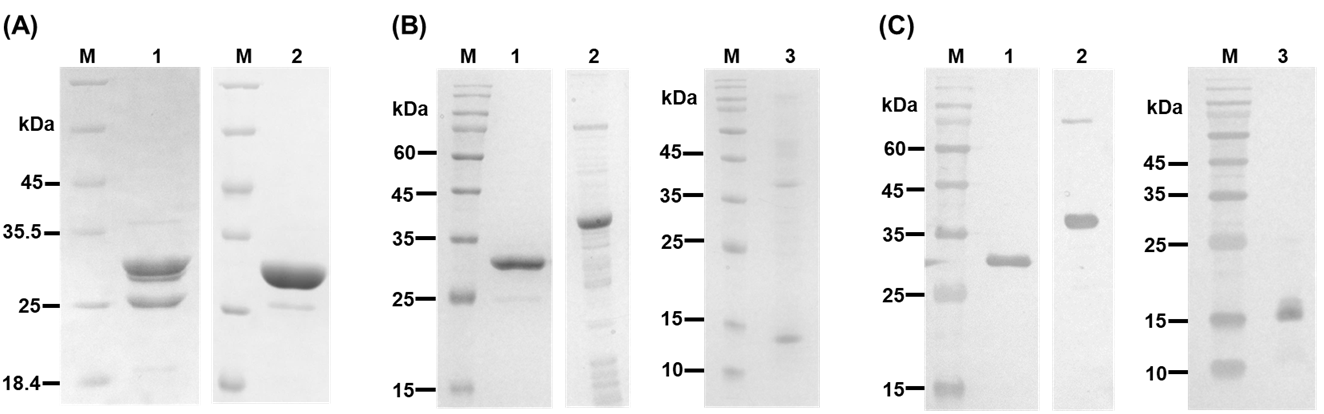
**
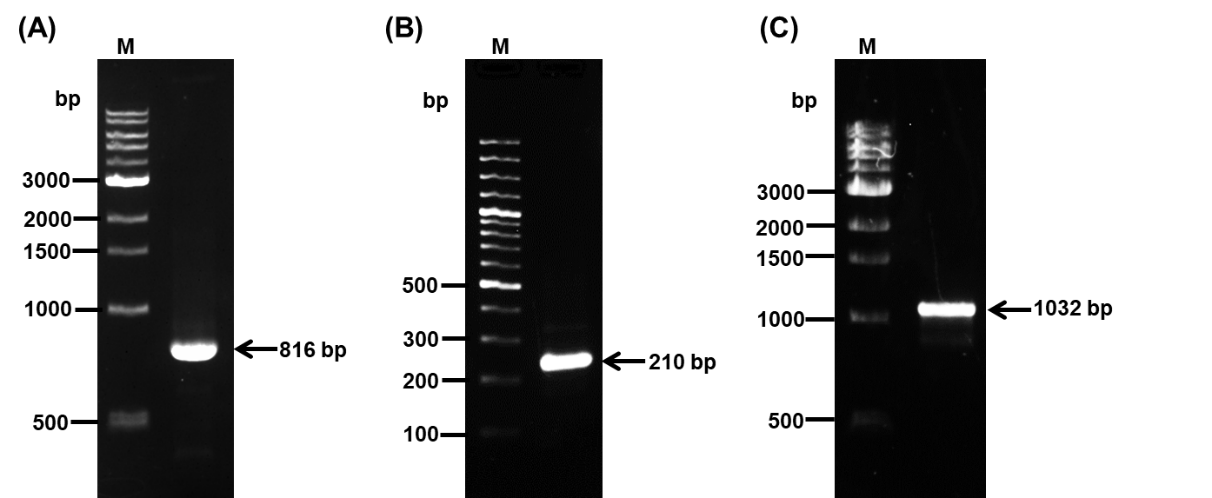
Figure S2.** Agarose gel electrophoresis. Full-length PCR products of (A) *bpss2242*, (B) *bpss2241*, and (C) *bpss2242+41*. Lane M, DNA ladder.

**Figure S3.** Analyses of purified recombinant proteins. (A) BPSS2242 was purified in the absence (lane 1) or presence (lane 2) of phenanthroline. (B) SDS-PAGE analysis and (C) Western blot analysis of purified BpSDRs. Lane M, molecular weight protein markers. Lane 1, BPSS2242; lane 2, BPSS2242+41 and lane 3, BPSS2241.

Comparison of the gene structure between *B. pseudomallei* K96243 and other *B. pseudomallei* isolates was performed and the result is shown in Supplementary Fig. S4.


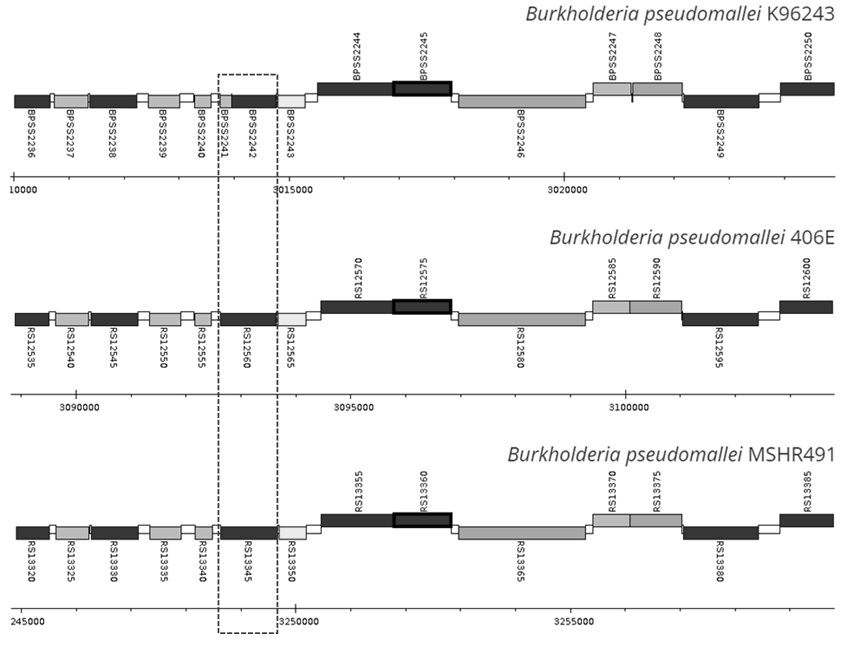


**Figure S4.** Comparison of *sdr* gene structure between *B. pseudomallei* isolate K96243 and other isolates. The *sdr* from other *B. pseudomallei* isolates composes of both *bpss2242* and *bpss2241* counterparts.

The raw image files of cropped gels and blots are shown below.


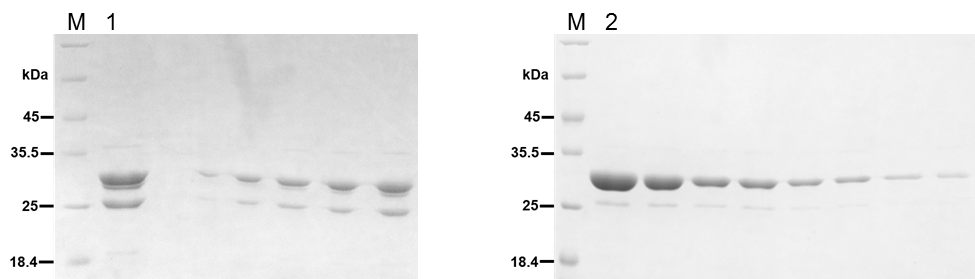


**Figure S5.** Raw image file of Figure S3A**.** SDS-PAGE analysis of BPSS2242 purified in the absence (lane 1) or presence (lane 2) of phenanthroline.


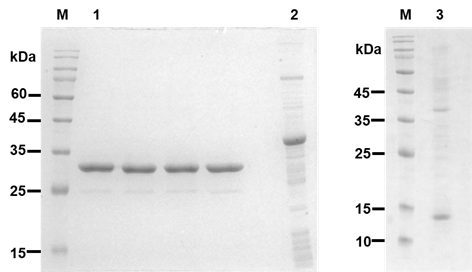


**Figure S6.** Raw image file of Figure S3B**.** SDS-PAGE analysis of purified BpSDRs. Lane M, molecular weight protein markers. Lane 1, BPSS2242; lane 2, BPSS2242+41 and lane 3, BPSS2241.


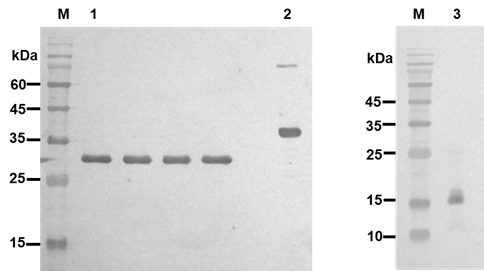


**Figure S7.** Raw image file of Figure S3C**.** Western blot analysis of purified BpSDRs. Lane M, molecular weight protein markers. Lane 1, BPSS2242; lane 2, BPSS2242+41 and lane 3, BPSS2241.


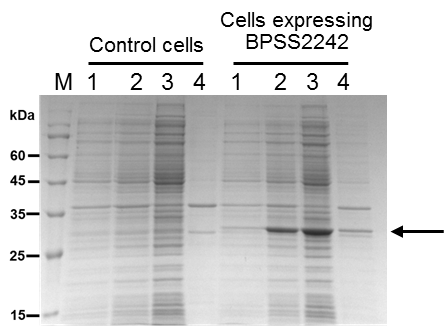


**Figure S8**. Raw image file of Figure 7A. Recombinant protein expression in BL21 (DE3). Lane M, molecular weight protein markers; lane 1, un-induced cells; lane 2, induced cells after 4 hours induction with 1 mM IPTG; lane 3, soluble fraction; lane 4, insoluble fraction. Control cells are *E. coli* BL21 (DE3) carrying empty pET23a plasmid. Control cells are *E. coli* BL21 (DE3) carrying empty pET23a plasmid.
